# More work for the same pay: trends in urethroplasty reimbursement from the medicare physician fee schedule (2013–2023)

**DOI:** 10.1007/s11255-026-05072-w

**Published:** 2026-03-03

**Authors:** Thriaksh Rajan, Abhav Garde, Kyle Scarberry, Robert Caleb Kovell, Ramy Abou Ghayda

**Affiliations:** 1 Case Western Reserve University School of Medicine, Cleveland, OH 44106 USA; 2 University Hospitals Ahuja Medical Center, 3999 Richmond Rd 3Rd Floor, Beachwood, OH 44122 USA; 3 Hospital of the University of Pennsylvania, Philadelphia, PA 19104 USA

**Keywords:** Urethroplasty, Urethral stricture, Medicare, Reimbursement, Health care economics

## Abstract

**Objectives:**

Urethroplasty is the gold standard for recurrent urethral stricture disease per the 2016 American Urological Association (AUA) guideline. Its sustainability depends on economic viability; however, recent payment cuts and inflationary pressures threaten reimbursement. This study analyzed: (1) trends in Medicare providers performing urethroplasty, (2) urethroplasty procedure volumes, and (3) nominal and inflation-adjusted Medicare reimbursement from 2013–2023, contextualized against comparator urologic procedures.

**Methods:**

This was a retrospective, cross-sectional study using the Centers for Medicare & Medicaid Services (CMS) Physician Fee Schedule and Medicare Part B Physician and Other Practitioners datasets from 2013–2023. Urethroplasty procedures were identified using CPT codes 53,400, 53,405, 53,410, 53,415, and 53,430. Comparator procedures included Cystourethroscopy with Direct Vision Internal Urethrotomy (DVIU, CPT 52276) and Endoscopic/Robotic-Assisted Radical Prostatectomy (CPT 55866). Reimbursement values were adjusted to August 2025 U.S. dollars using the Consumer Price Index.

**Results:**

Urethroplasty provider numbers and procedure volumes increased 33–45% and 36–65%, respectively. Nominal urethroplasty reimbursement remained stable (–2.5% to + 22.8%), while inflation-adjusted reimbursement declined 18–25% across all types. By contrast, DVIU demonstrated declining provider participation (–33.9%) and procedure volumes (–46.5%), with inflation-adjusted reimbursement declining 27.4%. Endoscopic prostatectomy showed growing provider participation (+ 33.4%) and volumes (+ 27.3%), but experienced the most severe nominal (–26.5%) and inflation-adjusted (–43.5%) reimbursement decline of all procedures examined.

**Conclusions:**

Urethroplasty faces a distinctive economic burden: rising clinical demand paired with stagnant nominal and substantially declining real reimbursement. While reimbursement erosion affects all urologic procedures studied, the combination of increasing utilization and inadequate payment adjustment is uniquely pronounced for reconstructive urology. Long-term sustainability will require reimbursement models that align payment with evidence-based, high-value surgical care.

## Introduction

Urethral stricture disease affects approximately 1% of the male population and disproportionately impacts older men who comprise the Medicare beneficiary population [1, 2]. This common condition results in significant morbidity, leading to numerous office visits, emergency department presentations, and hospital admissions that represent a substantial burden on the healthcare system ($191 million USD in 2000) [1, 3]. In 2016, the American Urological Association (AUA) released clinical guidelines recommending urethroplasty over repeated endoscopic procedures such as urethral dilation or direct visual internal urethrotomy (DVIU) for recurrent urethral strictures, based on superior long-term success rates and durability [4, 5]. Urethroplasty has since emerged as the gold standard for definitive management of stricture disease, offering patients a durable solution that reduces the need for repeated interventions and improves quality of life [6]. Given the prevalence of stricture disease in the Medicare population and the technical complexity of reconstructive surgery, urethroplasty represents a critical component of urologic practice that ensures access to specialized care for Medicare patients requiring definitive surgical management [7]. The clinical and economic viability of maintaining expertise in complex reconstructive urology depends on adequate reimbursement that reflects the specialized training, operative time, and technical skill required for these procedures [8, 9].

Urologists across the United States are facing mounting economic pressures, with physician reimbursement declining by 47.44% when adjusted for inflation from 2015 to 2024, while practice costs including staff salaries, equipment, facility expenses, and liability insurance continue to rise [10]. These financial challenges have been compounded by regulatory burden, administrative complexity, and persistent uncertainty in Medicare payment policy that undermines long-term practice planning [11]. In 2025, the Medicare conversion factor is set to decrease by approximately 2.83% from $33.2875 to $32.3465, driven by the expiration of the temporary conversion factor increase that Congress passed in March, with CMS lacking statutory authority to eliminate or reduce this cut independently [12]. Complex reconstructive procedures such as urethroplasty are suspected to be highly affected by this cut, as these operations require longer operative times, specialized equipment, and extensive postoperative care compared to endoscopic alternatives [13]. Without adequate reimbursement that accounts for inflation and the true costs of providing specialized surgical care, urologists may be unable to sustain reconstructive practices, potentially creating access barriers for Medicare beneficiaries who need definitive surgical management. Preserving economic viability for urethroplasty is essential not only for individual practices but for ensuring that patients can receive guideline-concordant care that offers superior long-term outcomes compared to repeated temporizing interventions.

While previous studies have examined general utilization trends for urethral stricture management, none have comprehensively analyzed Medicare reimbursement trends for urethroplasty across different procedure types over an extended time period spanning the 2016 AUA guideline publication [14, 15]. Therefore, the aims of this study were: 1) to analyze trends in the number of Medicare providers performing urethroplasty procedures from 2013 to 2023, 2) to examine trends in the volume of urethroplasty procedures performed during this period, and 3) to evaluate trends in both nominal and inflation-adjusted Medicare reimbursement for urethroplasty across different procedure types. This study provides concrete evidence that can inform multiple stakeholders: first, it equips the AUA and other specialty societies with data to advocate for elimination of statutory conversion factor cuts and pursue permanent payment reform through legislative action; second, it offers private practice urologists who provide urethroplasty critical information for negotiating contracts, setting financial expectations, and making strategic decisions about practice sustainability; and third, it enables urologists to recognize economic differences among various urethroplasty approaches, informing clinical decision-making for Medicare beneficiaries.

## Methods

### Study design and setting

This was a retrospective cross-sectional study of Medicare reimbursement and utilization for urethroplasty from 2013 through 2023. Procedures were identified using Current Procedural Terminology (CPT) codes: 53,400 (Urethroplasty, 2-stage reconstruction or repair of prostatic or membranous urethra; first stage), 53,405 (Urethroplasty, 2-stage reconstruction or repair of prostatic or membranous urethra; second stage), 53,410 (Urethroplasty, 1-stage reconstruction of male anterior urethra), 53,415 (Urethroplasty, transpubic or perineal, 1-stage, for reconstruction or repair of prostatic or membranous urethra), and 53,430 (Urethroplasty, reconstruction of female urethra). CPT codes 53,420 (Urethroplasty, 2-stage reconstruction or repair of membranous urethra; first stage) and 53,425 (Urethroplasty, 2-stage reconstruction or repair of membranous urethra; second stage) had no data available in the Medicare database and were therefore excluded from analysis; the absence of data for these codes likely reflects the infrequency of this specific surgical approach based on anatomical considerations and outcomes literature [16].

### Reimbursement data

Annual reimbursement values were extracted from the Centers for Medicare & Medicaid Services (CMS) Physician Fee Schedule (PFS) Look-up Tool [17]. Reimbursement was calculated using the standard formula defined by CMS with non-facility rates: Payment rate = Conversion factor × Total RVU. This extends to Total RVU = (work RVU × work GPCI) + (practice expense RVU × practice expense GPCI) + (malpractice RVU × malpractice GPCI), where RVUs represent the relative value units assigned to each CPT code and GPCIs represent the geographic practice cost indices. Conversion factors were applied annually at the national level. Nominal annual payment rates were collected as "non-adjusted values." To present changes in real terms, values were inflation-adjusted using the Consumer Price Index for All Urban Consumers (CPI-U) published by the U.S. Bureau of Labor Statistics [18]. All reimbursements are reported in August 1, 2025 U.S. dollars. Standard deviation (SD) for national reimbursement values were estimated by aggregating unweighted state-level PFS data.

### Utilization data

Utilization data were obtained from the Medicare Part B Physician & Other Practitioners – by Geography and Service dataset [19]. The primary utilization metric was the "Number of distinct Medicare beneficiary/per day services." Because the CPT codes included in this study represent primary urethroplasty procedures rather than revision or multi-service events, this metric was treated as equivalent to the number of unique beneficiaries receiving each procedure per year. The "Number of providers within HCPCS code and place of service" was also extracted to evaluate trends in the urethroplasty workforce and whether utilization was concentrated among a limited number of providers.

### Comparator data

To contextualize urethroplasty reimbursement trends within the broader urologic landscape, data were additionally collected for two comparator procedures: Cystourethroscopy with Direct Vision Internal Urethrotomy (DVIU, CPT 52276) and Endoscopic/Robotic-Assisted Radical Prostatectomy (CPT 55866). DVIU was selected as a direct clinical comparator, representing the endoscopic alternative to urethroplasty for urethral stricture management that the 2016 AUA guidelines recommend against for recurrent strictures. Endoscopic prostatectomy was selected as a non-reconstructive urologic control procedure, representing a high-volume, technically complex urologic surgery that serves as a within-specialty benchmark for reimbursement trends unrelated to stricture management. Identical data extraction methods, reimbursement calculations, and inflation-adjustment procedures were applied to comparator procedures as described above for urethroplasty.

### Statistical analysis

Descriptive statistics were calculated for each procedure type to summarize central tendencies and variability in reimbursement, utilization, and provider counts. Compound annual growth rates (CAGR) were computed using the formula: CAGR = (Ending value ÷ Beginning value)^(1 ÷ Number of years) – 1. Percentage change was calculated as: [(Ending value – Beginning value) ÷ Beginning value] × 100. To formally compare inflation-adjusted reimbursement trends across procedure types, two complementary statistical approaches were employed. First, simple linear regression models with year as the predictor variable were fitted separately for each procedure to estimate the annual rate of inflation-adjusted reimbursement decline (slope, R^2^, and p-value). Second, pairwise interaction models were constructed by pooling annual inflation-adjusted reimbursement data for endoscopic prostatectomy (reference group) and each comparator procedure individually into a linear regression with procedure type, year, and a procedure × year interaction term. The interaction term coefficient tests whether the rate of reimbursement decline (slope over time) of each procedure differs significantly from that of endoscopic prostatectomy. Endoscopic prostatectomy was designated as the reference group as it represented the non-reconstructive urologic control and demonstrated the steepest absolute rate of nominal and inflation-adjusted reimbursement decline of all procedures examined. Given the observational nature of the dataset and the limited sample size, analyses were intended to describe trends and associations rather than establish causal inference. All analyses were conducted using R (version 4.3.1; Vienna, Austria) by study team members with advanced training in biostatistics and health services research.

### Ethics statement

This study was exempt from institutional review board review and informed consent requirements as it did not involve human participants and relied exclusively on publicly available data.

## Results

### National volume of medicare urethroplasty providers (2013–2023)

**Fig. 1:**
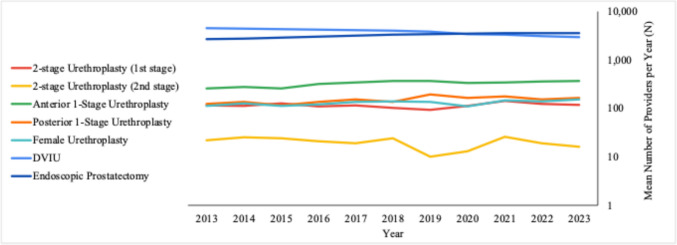
Mean number of medicare providers performing urethroplasty by procedure type (2013–2023)

The number of Medicare providers performing urethroplasty procedures demonstrated growth across most procedure types from 2013 to 2023 **(**Fig. 1**, **Table 1**).** Anterior 1-stage urethroplasty (CPT 53410) providers showed the most substantial increase, growing from 255 to 370 providers, representing a 45.10% change and a compound annual growth rate (CAGR) of 3.79% (mean 325.5 ± 43.02 providers). Posterior 1-stage urethroplasty (CPT 53415) providers increased from 124 to 165, a 33.06% change with a CAGR of 2.90% (mean 150.1 ± 23.44 providers). Female urethroplasty (CPT 53430) providers grew from 113 to 153, demonstrating a 35.40% change and CAGR of 3.08% (mean 130.2 ± 14.8 providers). Two-stage urethroplasty first stage (CPT 53400) providers showed minimal growth from 115 to 117, representing a 1.74% change and CAGR of 0.17% (mean 115.1 ± 12.7 providers). In contrast, two-stage urethroplasty second stage (CPT 53405) providers declined from 22 to 16, representing a −27.27% change and CAGR of −3.13% (mean 19.9 ± 5.15 providers). The number of Medicare providers performing DVIU declined substantially from 4,456 in 2013 to 2,944 in 2023, representing a –33.93% change and CAGR of –4.06% (mean 3,810.2 ± 536.44 providers). The number of Medicare providers performing endoscopic prostatectomy grew from 2,679 in 2013 to 3,574 in 2023, a + 33.41% change and CAGR of + 2.92% (mean 3,216.9 ± 345.40 providers).

**Table 1:** Medicare utilization and reimbursement characteristics for urethroplasty (2013–2023)

Category	Mean	SD	Change (%)	CAGR (%)
2-stage Urethroplasty (1st stage), CPT 53400				
Providers (N)	115.1	12.7	1.74%	0.17%
Procedures (N)	176.6	23.9	23.38%	2.12%
Reimbursement ($)	725.4	42.4	22.80%	2.07%
Inflation-Adjusted Reimbursement ($)	933.0	35.8	−5.48%	−0.56%
2-stage Urethroplasty (2nd stage), CPT 53405				
Providers (N)	19.9	5.2	−27.27%	−3.13%
Procedures (N)	24.6	7.5	−17.39%	−1.89%
Reimbursement ($)	822.7	69.2	−2.46%	−0.25%
Inflation-Adjusted Reimbursement ($)	1062.3	119.9	−24.92%	−2.83%
Anterior 1-stage Urethroplasty, CPT 53410				
Providers (N)	325.5	43.0	45.10%	3.79%
Procedures (N)	695.8	129.1	65.22%	5.15%
Reimbursement ($)	882.9	20.7	6.11%	0.59%
Inflation-Adjusted Reimbursement ($)	1138.9	78.9	−18.33%	−2.00%
Posterior 1-Stage Urethroplasty, CPT 53415				
Providers (N)	150.1	23.4	33.06%	2.90%
Procedures (N)	310.0	52.3	49.56%	4.11%
Reimbursement ($)	982.6	29.9	−1.76%	−0.18%
Inflation-Adjusted Reimbursement ($)	1270.0	121.4	−24.38%	−2.76%
Female Urethroplasty, CPT 53430				
Providers (N)	130.2	14.8	35.40%	3.08%
Procedures (N)	180.8	20.7	36.18%	3.14%
Reimbursement ($)	876.7	37.3	−1.20%	−0.12%
Inflation-Adjusted Reimbursement ($)	1133.3	114.6	−23.95%	−2.70%
Direct Visual Internal Urethrotomy, CPT 52276				
Providers (N)	3,810.2	536.4	−33.93%	−4.06%
Procedures (N)	9,839.2	2,086.8	−46.48%	−6.06%
Reimbursement ($)	335.1	16.6	−5.89%	−0.61%
Inflation-Adjusted Reimbursement ($)	433.5	48.9	−27.43%	−3.16%
Endoscopic Prostatectomy, CPT 55866				
Providers (N)	3,216.9	345.4	33.41%	2.92%
Procedures (N)	23,110.9	2,906.8	27.28%	2.44%
Reimbursement ($)	1,136.0	112.1	−26.54%	−3.04%
Inflation-Adjusted Reimbursement ($)	1,474.6	239.3	−43.46%	−5.54%

### National volume of medicare urethroplasty procedures (2013–2023)

**Fig. 2:**
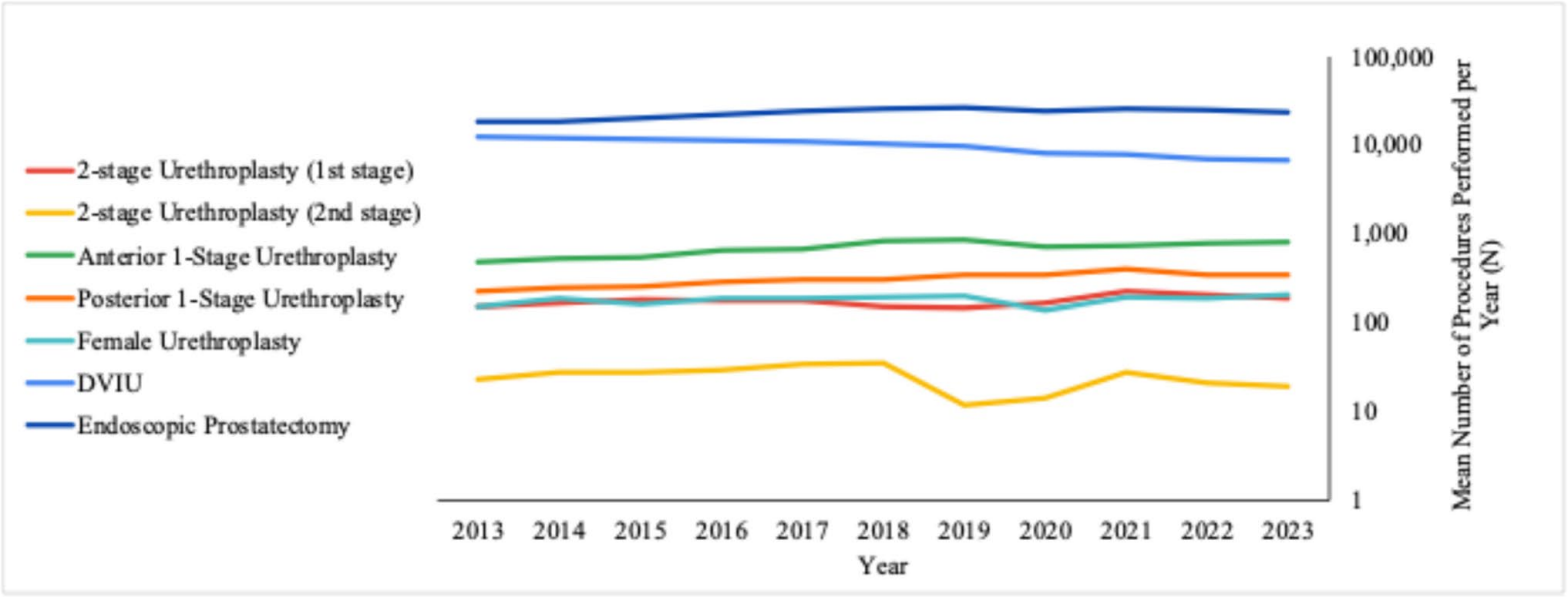
Mean annual volume of medicare urethroplasty procedures by type (2013–2023)

The volume of urethroplasty procedures performed increased substantially for most procedure types from 2013 to 2023 **(**Fig. 2**, **Table 1**).** Anterior 1-stage urethroplasty (CPT 53410) demonstrated the greatest growth, increasing from 483 to 798 procedures, representing a 65.22% change and CAGR of 5.15% (mean 695.8 ± 129.09 procedures). Posterior 1-stage urethroplasty (CPT 53415) procedures increased from 228 to 341, a 49.56% change with a CAGR of 4.11% (mean 310.0 ± 52.29 procedures). Female urethroplasty (CPT 53430) procedures grew from 152 to 207, representing a 36.18% change and CAGR of 3.14% (mean 180.8 ± 20.77 procedures). Two-stage urethroplasty first stage (CPT 53400) increased from 154 to 190 procedures, a 23.38% change with a CAGR of 2.12% (mean 176.6 ± 23.89 procedures). Two-stage urethroplasty second stage (CPT 53405) declined from 23 to 19 procedures, representing a −17.39% change and CAGR of −1.89% (mean 24.6 ± 7.54 procedures). Procedure volumes for DVIU similarly declined from 12,486 to 6,682, a –46.48% change with a CAGR of –6.06% (mean 9,839.2 ± 2,086.84 procedures). Procedure volumes for endoscopic prostatectomy increased from 18,447 to 23,479, representing a + 27.28% change with a CAGR of + 2.44% (mean 23,110.9 ± 2,906.83 procedures).

### National medicare reimbursement in urethroplasty (2013–2023)

**Fig. 3:**
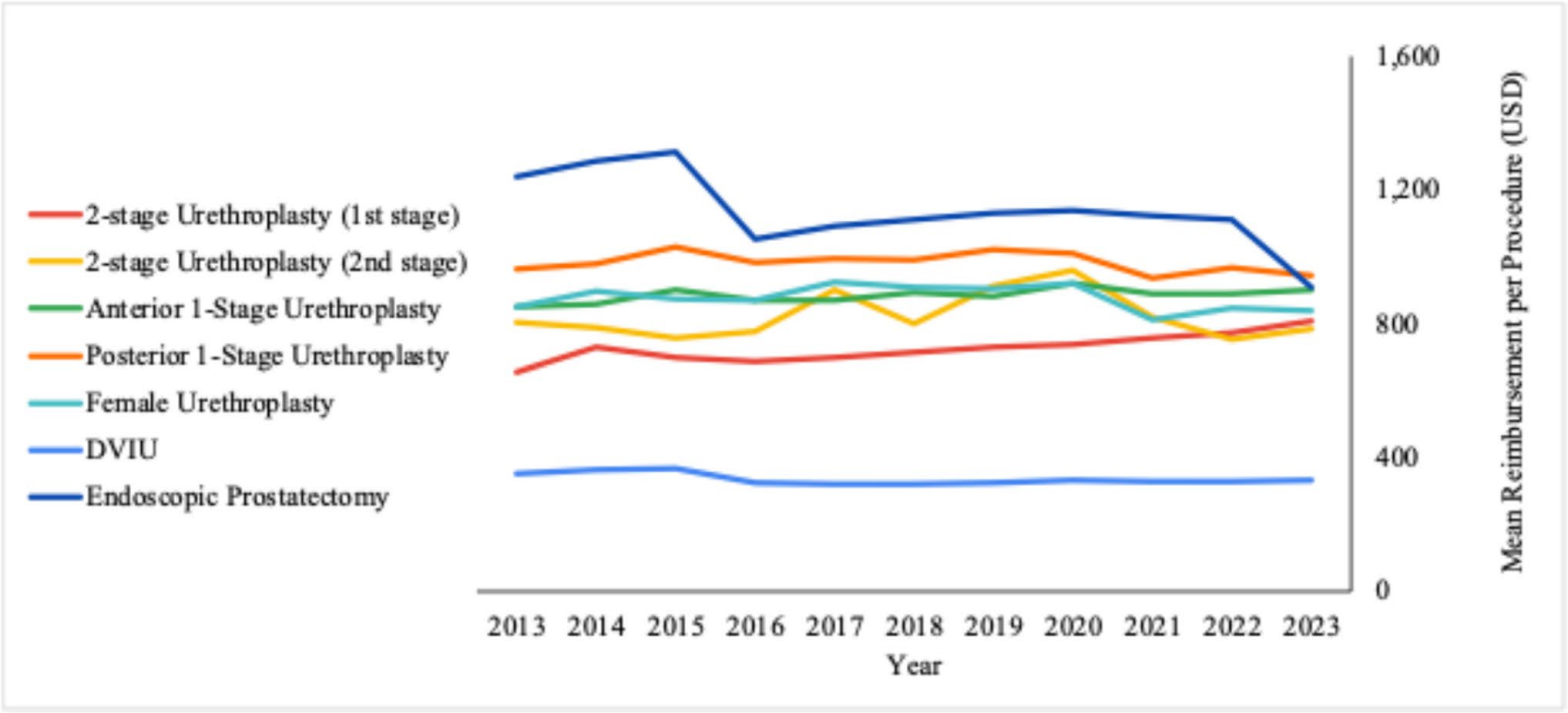
Mean medicare reimbursement per urethroplasty procedure by type (2013–2023)

Mean reimbursement per procedure remained relatively stable or showed minimal decline across most urethroplasty types from 2013 to 2023 **(**Fig. 3**, **Table 1**).** Two-stage urethroplasty first stage (CPT 53400) reimbursement increased from $656.44 to $806.08, representing a 22.80% change and CAGR of 2.07% (mean $725.4 ± $42.36). Anterior 1-stage urethroplasty (CPT 53410) reimbursement increased modestly from $848.58 to $900.39, a 6.11% change with a CAGR of 0.59% (mean $882.9 ± $20.7). Posterior 1-stage urethroplasty (CPT 53415) reimbursement decreased slightly from $961.48 to $944.55, representing a −1.76% change and CAGR of −0.18% (mean $982.6 ± $29.9). Female urethroplasty (CPT 53430) reimbursement declined from $849.88 to $839.69, a −1.20% change with a CAGR of −0.12% (mean $876.7 ± $37.25). Two-stage urethroplasty second stage (CPT 53405) showed a −2.46% change from $803.38 to $783.61 with a CAGR of −0.25% (mean $822.7 ± $69.2). DVIU nominal reimbursement remained relatively stable, declining modestly from $350.43 to $329.78, a –5.89% change with a CAGR of –0.61% (mean $335.1 ± $16.63). In contrast to the utilization growth, nominal reimbursement for endoscopic prostatectomy declined markedly from $1,237.90 to $909.37, a –26.54% change with a CAGR of –3.04% (mean $1,136.0 ± $112.09).

### National inflation-adjusted medicare reimbursement in urethroplasty (2013–2023)

**Fig. 4:**
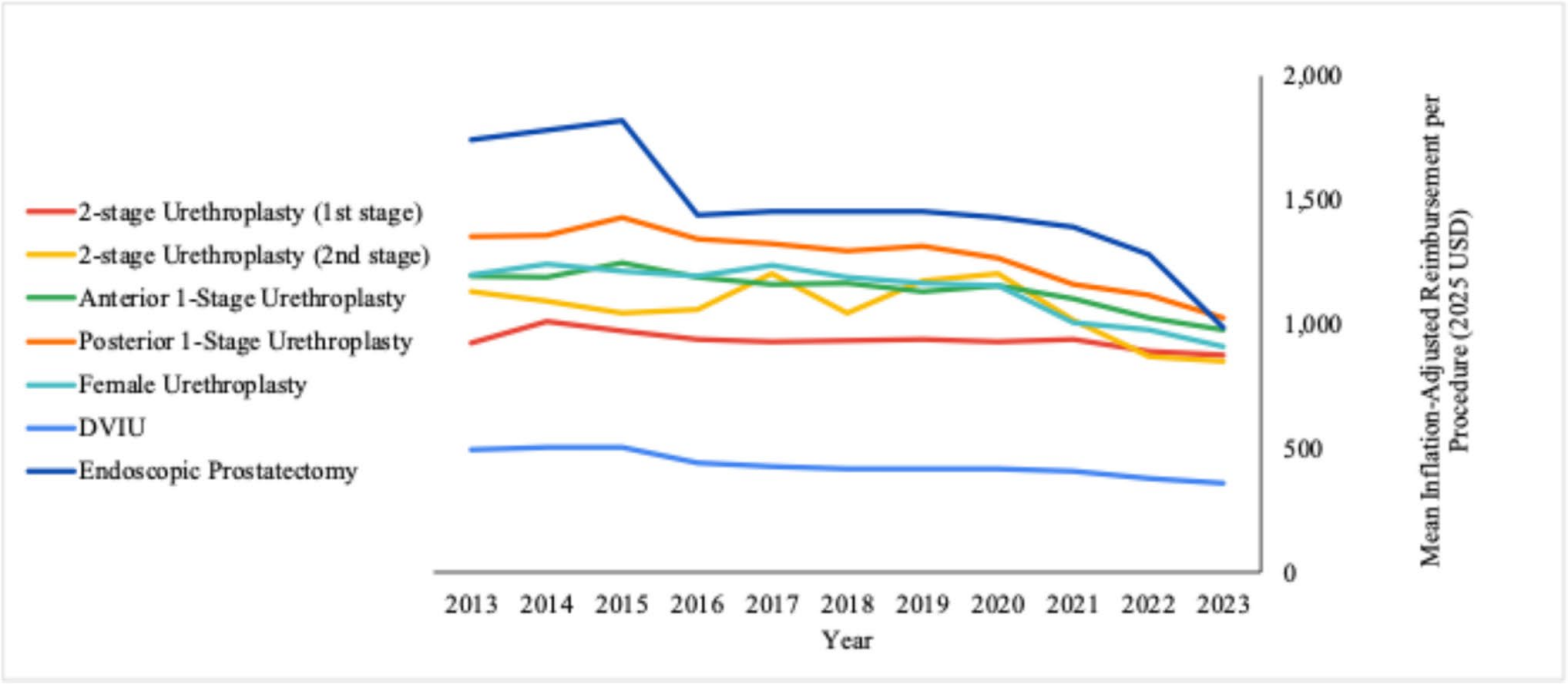
Mean inflation-adjusted medicare reimbursement per urethroplasty procedure by type (2013–2023, adjusted to 2025)

When adjusted for inflation in 2025, mean reimbursement per procedure declined substantially across all urethroplasty types from 2013 to 2023 **(**Fig. 4**, **Table 1**).** Posterior 1-stage urethroplasty (CPT 53415) demonstrated the largest inflation-adjusted decline, decreasing from $1,352.69 to $1,022.87, representing a −24.38% change and CAGR of −2.76% (mean $1,270.0 ± $121.42). Two-stage urethroplasty second stage (CPT 53405) declined from $1,130.26 to $848.58, a −24.92% change with a CAGR of -2.83% (mean $1,062.3 ± $119.89). Female urethroplasty (CPT 53430) decreased from $1,195.68 to $909.31, representing a −23.95% change and CAGR of −2.70% (mean $1,133.3 ± $114.59). Anterior 1-stage urethroplasty (CPT 53410) declined from $1,193.85 to $975.05, an −18.33% change with a CAGR of −2.00% (mean $1,138.9 ± $78.94). Two-stage urethroplasty first stage (CPT 53400) showed the smallest decline from $923.53 to $872.92, a −5.48% change with a CAGR of −0.56% (mean $933.0 ± $35.82). DVIU inflation-adjusted reimbursement declined from $493.01 to $357.78, representing a –27.43% change and CAGR of –3.16% (mean $433.5 ± $48.95). Inflation-adjusted reimbursement for endoscopic prostatectomy declined from $1,741.57 to $984.77, representing the most severe decline of any procedure examined at –43.46% and a CAGR of –5.54% (mean $1,474.6 ± $239.32).

### Comparative context of urethroplasty and DVIU to endoscopic prostatectomy

Individual linear regression confirmed statistically significant downward trends in inflation-adjusted reimbursement for all procedures except two-stage urethroplasty second stage (CPT 53405; slope: –$19.43/yr, R^2^ = 0.289, p = 0.088), which showed a non-significant negative trend, likely reflecting the small sample size and high variability inherent to this low-volume procedure. Endoscopic prostatectomy demonstrated the steepest rate of decline of all procedures examined (slope: –$64.48/yr, R^2^ = 0.798, p < 0.001), followed by posterior 1-stage urethroplasty (–$32.60/yr, p < 0.001), female urethroplasty (–$29.58/yr, p < 0.001), anterior 1-stage urethroplasty (–$20.71/yr, p < 0.001), DVIU (–$13.91/yr, p < 0.001), and two-stage urethroplasty first stage (–$7.59/yr, p < 0.05).

Pairwise interaction models with endoscopic prostatectomy as the reference group revealed that all comparator procedures declined at a significantly slower rate than prostatectomy (all p < 0.05) **(**Table 2**)**. DVIU and two-stage urethroplasty first stage showed the smallest rates of decline and the largest slope differences from prostatectomy (DVIU: + $50.57/yr difference, p < 0.001; CPT 53400: + $56.88/yr, p < 0.001). Two-stage urethroplasty second stage (+ $45.04/yr, p < 0.01) and anterior 1-stage urethroplasty (+ $43.77/yr, p < 0.01) showed intermediate differences, while posterior 1-stage urethroplasty (+ $31.87/yr, p < 0.05) and female urethroplasty (+ $34.89/yr, p < 0.05) demonstrated the smallest slope differences from prostatectomy, indicating their rates of decline most closely approximated the reference procedure. Importantly, the finding that all procedures declined significantly slower than prostatectomy does not diminish the clinical significance of urethroplasty reimbursement erosion; rather, it contextualizes prostatectomy as experiencing an unusually severe decline driven by targeted CMS RVU revaluation, while urethroplasty undergoes a slower but sustained and compounding erosion in the setting of rising procedure volumes.

**Table 2:** Linear regression comparison of inflation-adjusted reimbursement between urethroplasty and DVIU to endoscopic prostatectomy

Procedure vs. Endoscopic Prostatectomy	Slope ($/yr)	Slope Difference	R^2^	*p*-value
DVIU (CPT 52276)	−13.91	+ 50.57	0.888	< 0.001
2-stage Urethroplasty 1st stage (CPT 53400)	−7.59	+ 56.88	0.494	< 0.001
2-stage Urethroplasty 2nd stage (CPT 53405)	−19.43	+ 45.04	0.289	0.006
Anterior 1-stage Urethroplasty (CPT 53410)	−20.71	+ 43.77	0.757	0.01
Posterior 1-stage Urethroplasty (CPT 53415)	−32.60	+ 31.87	0.793	0.03
Female Urethroplasty (CPT 53430)	−29.58	+ 34.89	0.733	0.02

## Discussion

Urethral stricture disease represents a common urologic condition that significantly impacts patient quality of life, and understanding reimbursement trends is essential for ensuring continued access to definitive surgical management. Despite growing recognition of urethroplasty as the gold standard treatment, little is known about how Medicare payment trends have evolved relative to clinical demand and inflation over the past decade. Our analysis reveals that while both provider numbers and procedure volumes have increased substantially across most urethroplasty types, nominal reimbursement has remained largely stagnant, and inflation-adjusted reimbursement has declined by 18–25% across all procedure types. These findings demonstrate that urologists are performing more complex reconstructive procedures with growing frequency yet receiving progressively less real compensation for this specialized work. AUA policymakers and surgeons should use these findings to advocate for reimbursement models that appropriately value complex surgical procedures and ensure continued access to specialized reconstructive urologic care for Medicare beneficiaries. Hospital administrators and specialty societies may intensify efforts to educate stakeholders about the economic pressures threatening the viability of reconstructive urology practices.

### National volume of medicare urethroplasty providers (2013–2023)

The substantial growth in provider numbers (33–45% for most procedure types) reflects increasing recognition of urethroplasty as definitive treatment and likely represents successful dissemination of the 2016 AUA guidelines recommending urethroplasty over repeated endoscopic procedures for recurrent strictures [4, 20]. Importantly, this workforce expansion also reflects deliberate structural investment in reconstructive urology training: Society of Genitourinary Reconstructive Surgeons (SGURS) fellowship programs expanded substantially over this same period, with Calvo et al. (2025) documenting that growth in fellowship training directly mirrors the evolution of the discipline, producing an increasing number of surgeons with dedicated reconstructive training entering practice [21]. Some might contend this growth could reflect practice expansion by existing providers rather than new surgeons entering the field, but the convergence of guideline publication, fellowship program expansion, and the magnitude of increase across multiple procedure types together suggests genuine and structurally grounded workforce expansion. Previous studies examining urethral stricture management trends have documented similar increases in urethroplasty utilization following guideline publication, supporting our findings [22, 23]. The particularly robust growth in anterior 1-stage urethroplasty providers (45.10%) likely reflects the relative technical accessibility of this approach, while the decline in two-stage second stage procedure providers (−27.27%) aligns with published reports suggesting these are reserved for the most complex cases and may be increasingly concentrated among high-volume reconstructive centers [16]. Training program directors may enhance fellowship training opportunities in reconstructive urology, recognizing that the fellowship pipeline itself is vulnerable to the reimbursement pressures documented here. Specialty societies can continue to advocate for reimbursement policies that reflect the specialized training required for these procedures.

### National volume of medicare urethroplasty procedures (2013–2023)

The substantial increase in procedure volumes (36–65% for most types) similarly demonstrates successful adoption of guideline-concordant care following the 2016 AUA recommendation favoring urethroplasty over repeated endoscopic interventions for recurrent strictures [4]. It may be reasoned that increasing volumes could reflect overutilization, but AUA research and the OPEN RCT have both demonstrated superior long-term outcomes and cost-effectiveness for urethroplasty compared to repeated endoscopic management [24, 25]. Studies examining management trends of urethral stricture disease have reported similar increases in urethroplasty utilization over comparable time periods, with growth rates ranging from 40–70% depending on procedure type and population studied [22, 23, 26]. The particularly robust growth in anterior 1-stage urethroplasty procedures (65.22%) reflects both the large proportion of anterior strictures in the overall stricture population and the technical feasibility of single-stage repairs. The modest growth in two-stage first stage procedures (23.38%) coupled with declining second stage procedures suggests surgeons increasingly favor single-stage approaches when clinically appropriate. Policymakers and payers may recognize this increasing demand as evidence of value-based care delivery.

### National medicare reimbursement in urethroplasty (2013–2023)

Nominal reimbursement showed stagnation for most procedures (ranging from −2.5% to + 22.8%), with most showing less than 7% change over the decade. Some healthcare economists might contend that stable nominal reimbursement represents acceptable payment policy during periods of low inflation, but this fails to account for practice expenses that consistently increase by 3–4% annually [27]. Studies examining surgical practice economics have documented that operating costs for complex procedural specialties substantially outpace general inflation, creating a widening gap between revenue and expenses [28]. Previous analyses of Medicare reimbursement trends across surgical specialties have reported similar patterns of stagnant nominal payments, with reconstructive subspecialties experiencing the most pronounced relative devaluation [29]. CMS and private payers may recognize that maintaining stable nominal reimbursement effectively devalues complex surgical procedures over time, potentially creating access barriers as fewer surgeons can sustain specialized reconstructive practices.

### National inflation-adjusted medicare reimbursement in urethroplasty (2013–2023)

The universal decline in inflation-adjusted reimbursement across all urethroplasty types (ranging from −5.5% to −24.9% when adjusted to August 1, 2025 dollars) represents a systematic devaluation of complex reconstructive surgery that threatens long-term sustainability of guideline-concordant care. Some might contend that improved surgical efficiency could justify lower inflation-adjusted reimbursement, but urethroplasty operative times and technical complexity have remained relatively constant while specialized training requirements have increased [8, 9]. Published analyses examining inflation-adjusted reimbursement trends across surgical specialties have documented similar patterns of real payment decline, with complex reconstructive procedures experiencing disproportionate erosion, suggesting our findings reflect broader systemic Medicare payment issues [29]. The severity of this decline is evident when comparing urethroplasty inflation-adjusted reimbursement CAGR (−0.56% to −2.83%) against the Federal CPI CAGR of 2.73% during the study period (CPI: 232.957 in 2013 to 304.702 in 2023), demonstrating that reimbursement declined substantially even as costs increased [18]. The 2016 AUA guidelines were based on superior outcomes and cost-effectiveness, yet our observed reimbursement trends suggest payment policy has not kept pace with evidence-based recommendations, creating misalignment between guidelines and financial incentives [4]. Furthermore, the impending 2.83% conversion factor cut by CMS will further compound these declines without legislative intervention such as the Medicare Patient Access and Practice Stabilization Act of 2024, which the AUA supports as part of ongoing advocacy for permanent conversion factor reform [12]. Policymakers should consider that cumulative 2–3% annual real declines compound over time to create the substantial 18–25% erosion observed, fundamentally altering the economic viability of reconstructive urology practices.

### Comparative context of urethroplasty and DVIU to endoscopic prostatectomy

The addition of DVIU and endoscopic prostatectomy as comparator procedures confirms that reimbursement erosion is a systemic phenomenon across urologic surgery, consistent with published analyses documenting real payment declines across surgical subspecialties [29]. Yet our formal statistical comparisons reveal that the magnitude and mechanism of decline differ meaningfully by procedure type, and that the specific burden facing urethroplasty is both distinct and underappreciated in the existing literature. Individual linear regression demonstrated significant downward trends across nearly all procedures, with endoscopic prostatectomy exhibiting the steepest rate of inflation-adjusted reimbursement decline (–$64.48/yr, R^2^ = 0.798, p < 0.001). Pairwise interaction models confirmed that every comparator procedure declined at a significantly slower rate than prostatectomy (all p < 0.05), with slope differences ranging from + $31.87/yr for posterior urethroplasty to + $56.88/yr for two-stage first stage urethroplasty.

This finding must be interpreted carefully rather than reassuringly. The severe erosion observed for prostatectomy was driven substantially by targeted CMS RVU revaluation—a discrete policy event producing a sharp single-year nominal decline of approximately 20% between 2015 and 2016—a mechanism distinct from the sustained inflationary erosion affecting urethroplasty [12, 30]. This pattern of abrupt procedure-specific RVU revaluation has been documented for robotic surgical codes broadly and raises a critical warning for urethroplasty: if CMS were to pursue analogous revaluation of reconstructive CPT codes, the compounding effect on an already-eroding reimbursement baseline could be severe [12, 29]. Prior analyses examining inflation-adjusted reimbursement across surgical specialties have similarly documented that reconstructive and technically complex subspecialty procedures face disproportionate cumulative erosion relative to higher-volume procedural counterparts [29]. The present study extends this literature by providing the first decade-long, procedure-specific comparative analysis within urology, demonstrating that urethroplasty's economic challenge is not merely a reflection of broad Medicare payment trends but is exacerbated by the unique combination of guideline-driven volume growth and payment stagnation.

DVIU demonstrated a significant but modest rate of decline (–$13.91/yr, p < 0.001) alongside sharp declines in provider participation (–33.9%) and procedure volumes (–46.5%), consistent with intended guideline-driven shifts away from endoscopic management documented in recent utilization studies [22, 23]. DVIU's economic and utilization trajectories are aligned with clinical guidelines while urethroplasty's are not. Specifically, growing volumes without commensurate reimbursement growth represents a fundamental misalignment between payment policy and evidence-based care that distinguishes this study's findings from prior work examining reimbursement trends in isolation. Legislative reform providing permanent inflation-indexed conversion factor updates, as supported by the AUA through the Medicare Patient Access and Practice Stabilization Act of 2024, will be critical to correct this misalignment before access barriers emerge for Medicare beneficiaries requiring definitive reconstructive care.

### Limitations

This study is not without limitations. Our analysis relies on Medicare claims data, which may not fully capture trends in younger populations or those with private insurance; however, Medicare beneficiaries constitute a substantial proportion of stricture patients given age-related incidence, making our findings highly relevant for this vulnerable population. Moreover, we did not account for potential changes in case complexity or comorbidity burden over time, which could influence work required; nonetheless, the CPT coding system is designed to account for procedural complexity. Our study period ends in 2023 and does not capture the 2025 conversion factor decrease; however, this limitation strengthens our central finding by suggesting reimbursement pressures will worsen without policy intervention. Additionally, the comparator procedures selected represent only a subset of urologic procedures and may not capture the full spectrum of reimbursement trends across the specialty. The marked nominal reimbursement decline observed for endoscopic prostatectomy reflects, in part, CMS RVU revaluation specific to robotic surgical codes and may not be directly generalizable to other procedure types. Despite these limitations, this study provides the first comprehensive decade-long analysis of Medicare urethroplasty reimbursement trends using national data, offering critical insights into the economic sustainability of providing complex reconstructive urologic care.

## Conclusion

In the summary, over the past decade, urologists have faced increasing clinical demand for urethroplasty procedures while experiencing substantial erosion in inflation-adjusted reimbursement, with real compensation declining by 18–25% across all procedure types despite growing provider numbers and volumes reflecting successful guideline implementation. Specialty societies, including the AUA, must intensify advocacy efforts to support the Medicare Patient Access and Practice Stabilization Act of 2024, which would eliminate the 2025 conversion factor cut and provide inflation-indexed updates. Hospital administrators and practice leaders should use these data when negotiating facility fees and employment contracts to ensure adequate compensation for the specialized training and technical complexity required for reconstructive procedures. Future studies should examine whether declining real reimbursement has created geographic disparities in access to urethroplasty using claims data linked to patient outcomes and travel distances to assess whether payment pressures have influenced surgical decision-making or quality of care.

## Data Availability

No datasets were generated or analysed during the current study.
